# Arg Deficiency Does not Influence the Course of Myelin Oligodendrocyte Glycoprotein (MOG35-55)-induced Experimental Autoimmune Encephalomyelitis

**DOI:** 10.4172/2155-9899.1000420

**Published:** 2016-05-23

**Authors:** Freja Aksel Jacobsen, Camilla Hulst, Thomas Bäckström, Anthony J. Koleske, Åsa Andersson

**Affiliations:** 1 Department of Drug Design and Pharmacology, Faculty of Health and Medical Sciences, University of Copenhagen, Copenhagen, Denmark; 2 Novo Nordisk A/S, Gentofte, Denmark; 3 Novo Nordisk A/S, Måløv, Denmark; 4 BTB Pharma, Malmö, Sweden; 5 Department of Molecular Biophysics and Biochemistry, Yale University School of Medicine, New Haven CT 06520, USA

**Keywords:** Abelson related gene, Experimental autoimmune encephalomyelitis, Lymphocyte phenotypes, Genotype frequency

## Abstract

**Background:**

Inhibition of Abl kinases has an ameliorating effect on the rodent model for multiple sclerosis, experimental autoimmune encephalomyelitis, and arrests lymphocyte activation. The family of Abl kinases consists of the Abl1/Abl and Abl2/Arg tyrosine kinases. While the Abl kinase has been extensively studied in immune activation, roles for Arg are incompletely characterized. To investigate the role for Arg in experimental autoimmune encephalomyelitis, we studied disease development in Arg^−/−^ mice.

**Methods:**

Arg^−/−^ and Arg^+/+^ mice were generated from breeding of Arg^+/−^ mice on the C57BL/6 background. Mice were immunized with the myelin oligodendrocyte glycoprotein (MOG)35–55 peptide and disease development recorded. Lymphocyte phenotypes of wild type Arg^+/+^ and Arg^−/−^ mice were studied by *in vitro* stimulation assays and flow cytometry.

**Results:**

The breeding of Arg^+/+^ and Arg^−/−^ mice showed skewing in the frequency of born Arg^−/−^ mice. Loss of Arg function did not affect development of experimental autoimmune encephalomyelitis, but reduced the number of splenic B-cells in Arg^−/−^ mice following immunization with MOG peptide.

**Conclusions:**

Development of MOG-induced experimental autoimmune encephalomyelitis is not dependent on Arg, but Arg plays a role for the number of B cells in immunized mice. This might suggest a novel role for the Arg kinase in B-cell trafficking or regulation. Furthermore, the results suggest that Arg is important for normal embryonic development.

## Introduction

Non-receptor tyrosine kinases are essential for immune cell activation and mediate signal transduction downstream of immune receptors [1]. The Abl1/Abl and Abl2/Arg tyrosine kinases comprise the Abl family of tyrosine kinases in vertebrates. Abl and Arg share the same domain structure in the N-terminal half of the proteins, and their tandem SH3, SH2, and kinase domain sequences are ~90% identical [1–3].

The two kinases are believed to have overlapping functions. Abl^−/−^ Arg^−/−^ double knockout mice die during embryonic development, while Abl^−/−^ and Arg^−/−^ single knockouts survive embryonic development even though prenatal mortality is higher in Abl^−/−^ mice compared to wild type (WT) mice [3,4]. Abl kinases are unique for their extended C-terminal parts and their ability to bind directly to, and organize, filamentous actin (F-actin) in the cytoskeleton [5]. In contrast to the N-terminus, Abl kinases differ greatly in domain structure in their C- terminal parts. Arg has two F-actin-binding domains and one microtubule-binding domain [6,7], and is a key player in regulation of cytoskeletal actin organization and modulation [8–10]. Roles for Abl in immune cell signaling have been broadly investigated [11–16], while the possible role for Arg is less studied. In contrast to Abl, Arg is also highly expressed in mature B-cells [17]. Inhibition of Abl kinases has an immunosuppressive effect. We (Jacobsen FA. et al., to be published), and others, have recently shown that the tyrosine kinase inhibitor, Imatinib, ameliorates disease progression, when tested in the murine model for multiple sclerosis, experimental autoimmune encephalomyelitis (EAE) [18–20]. To investigate the role for Arg in EAE pathogenesis, we induced disease in a mouse strain, where the Abl2/Arg gene had been deleted. Here we show that no differences in disease susceptibility, progression, and severity, were observed between Arg^+/+^ and Arg^−/−^ mice, when tested in the myelin oligodendrocyte glycoprotein peptide 35–55 (MOG35–55)-induced EAE model. Loss of Arg function led, however, to a significant reduction in the relative number of splenic B cells in immunized Arg^−/−^ mice.

## Materials and Methods

### Animals and genotyping

Arg^−/−^ mice were generated in a 129Sv/J x C57BL/6 mixed genetic background (Department of Molecular Biophysics and Biochemistry, Yale University School of Medicine, New Haven, CT, USA) as previously described [3], and were backcrossed to the C57BL/6J strain obtained from the Jackson Laboratory (Bar Harbor, ME). Mice were housed at the Department of Experimental Medicine, Faculty of Health and Medical Sciences, University of Copenhagen, Denmark. Genotypes were determined by PCR on purified DNA from tail tissue (mix of three primers: 5’-AAGGGCATCTCTAATTGTAAG GAGGAAGG-3’, 5’-CTGCAGTGCAACCCACGTGTGGGGA-3’, and 5’-AATTGACCTGCAGGGGCCCTCGAGG-3’), followed by separation on a 1.8% agarose gel by electrophoresis.

### Induction and evaluation of EAE

Arg^+/+^ and Arg^−/−^ mice were immunized subcutaneously at the base of the tail with 150 μg MOG_35–55_ (Schafer, Denmark) in PBS emulsified 1:1 in Freund’s incomplete adjuvant (IFA) (Sigma-Aldrich, St. Louis, MO) with 400 μg heat-inactivated mycobacteria tuberculosis H37Ra (DIFCO laboratories, Detroit, MI). Peritoneal injections of pertussis toxin from *Bordetella pertussis* (Sigma-Aldrich, St. Louis, MO) (400 ng) in PBS were administrated to mice at the day of immunization and two days post immunization. Mice were observed for disease onset and progression monitored by scoring: (0) No clinical symptoms; (1) loss of tail tonus; (2) mild paresis in one or both hind legs; (3) moderate paresis in one or both hind legs; (4) severe paresis in one or both hind legs; (5) paresis in one or both hind legs and any significant paresis in front leg; (6) moribund or diseased. In accordance with predefined human endpoints and approved study protocol (Animal Experiments Inspectorate permission number: 2010/561–1920), mice receiving a score above 4, or having lost more than 20% of their pre-study body mass, were euthanized.

### Preparation of cells and flow cytometry

Single-cell suspensions from spleen and inguinal lymph nodes were prepared in complete culture medium (Dulbecco’s modified eagle’s medium + GlutaMAX-I, 1 % fetal bovine serum, 1 mM Hepes, 5 μM 2- mercaptoethanol, and penicillin/streptomycin (50,000 units/10,000 μg)). All reagents were purchased from Invitrogen, Thermo Fisher Scientific, Waltham, MA. Red blood cells were lysed (BD Pharm Lysing Buffer, BD Biosciences, San Jose, CA) in spleen samples. Splenic and inguinal lymph node lymphocytes from naïve and MOG_35_-_55_ immunized mice were stained with anti-mouse CD4-phycoerythrin (PE) (BD Biosciences, San Jose, CA), anti-mouse CD8-PE-cyanine5 (CY5) (BD Biosciences, San Jose, CA), and anti-mouse CD19- fluorescein isothiocyanate (FITC) (BD Pharmingen, San Diego, CA), and fixed with Cytofix Fixation Buffer (BD Biosciences, San Jose, CA) for measurements on a FACS Calibur (BD Biosciences, San Jose, CA) or Gallios Flow Cytometer (Beckman Coulter, Brea, CA). Data was analyzed with the FlowJo soware.

### Proliferation assay

Splenic lymphocytes (2 × 10^5^ cells/well) from naïve Arg^+/+^ and Arg^−/−^ mice were stimulated *in vitro* with purified anti-mouse CD3 (BD Biosciences, San Jose, CA) and anti-mouse CD28 (eBioscience, San Diego, CA), Concanavalin A (ConA) (Sigma-Aldrich, St. Louis, MO), lipopolysaccharide (LPS) (Sigma-Aldrich, St. Louis, MO), and F(ab’)2 Fragment Goat Anti-Mouse IgM (Jackson Immuno Research, West Grove, PA). Cells were stimulated for 72 hours, and pulsed with [3H]- thymidine (1 μCi/well) (Perkin Elmer, Waltham, MA) for the last 16–18 hours. Cells were harvested and [3H]-thymine incorporation monitored on a TopCount NXT (Perkin Elmer, Waltham, MA).

### Cytokine assay

Splenic lymphocytes (2 × 10^5^ cells/well) from naïve Arg^+/+^ and Arg^−/−^ mice were stimulated *in vitro* with purified anti-mouse CD3 (BD Biosciences, San Jose, CA) and anti-mouse CD28 (eBioscience, San Diego, CA). Supernatant was collected at 48 hours of stimulation and IL-2 concentration determined by ELISA using purified rat anti‐mouse IL‐2 (JES6–1A1 2), Biotin rat anti‐mouse IL‐2 (JES6–5H4), recombinant mouse IL‐2, and Avidin‐Horseradish Peroxidase (HRP). All reagents were purchased from BD Biosciences, San Jose, CA.

### Statistics

Statistical calculations were done with Mann-Whitney test in GraphPad Prism.

## Results

### Arg affects embryonic development

To study the role for the tyrosine kinase Arg in CNS inflammation and activation of immune cells, breeding of mice heterozygous for Arg (Arg^+/−^) on a C57BL/6 genetic background, which is susceptible for EAE upon immunization with MOG35–55 peptide, was set up. The genotype ratio of the progeny from the heterozygous breeding revealed that the frequency of born Arg^−/−^ pups was only 10% of the total number of offspring, which should be compared to the expected 25% (Table 1). This shows that loss of Arg is not compatible with normal embryonic development.

### EAE-progression Arg^−/−^ mice

**Figure 1:**
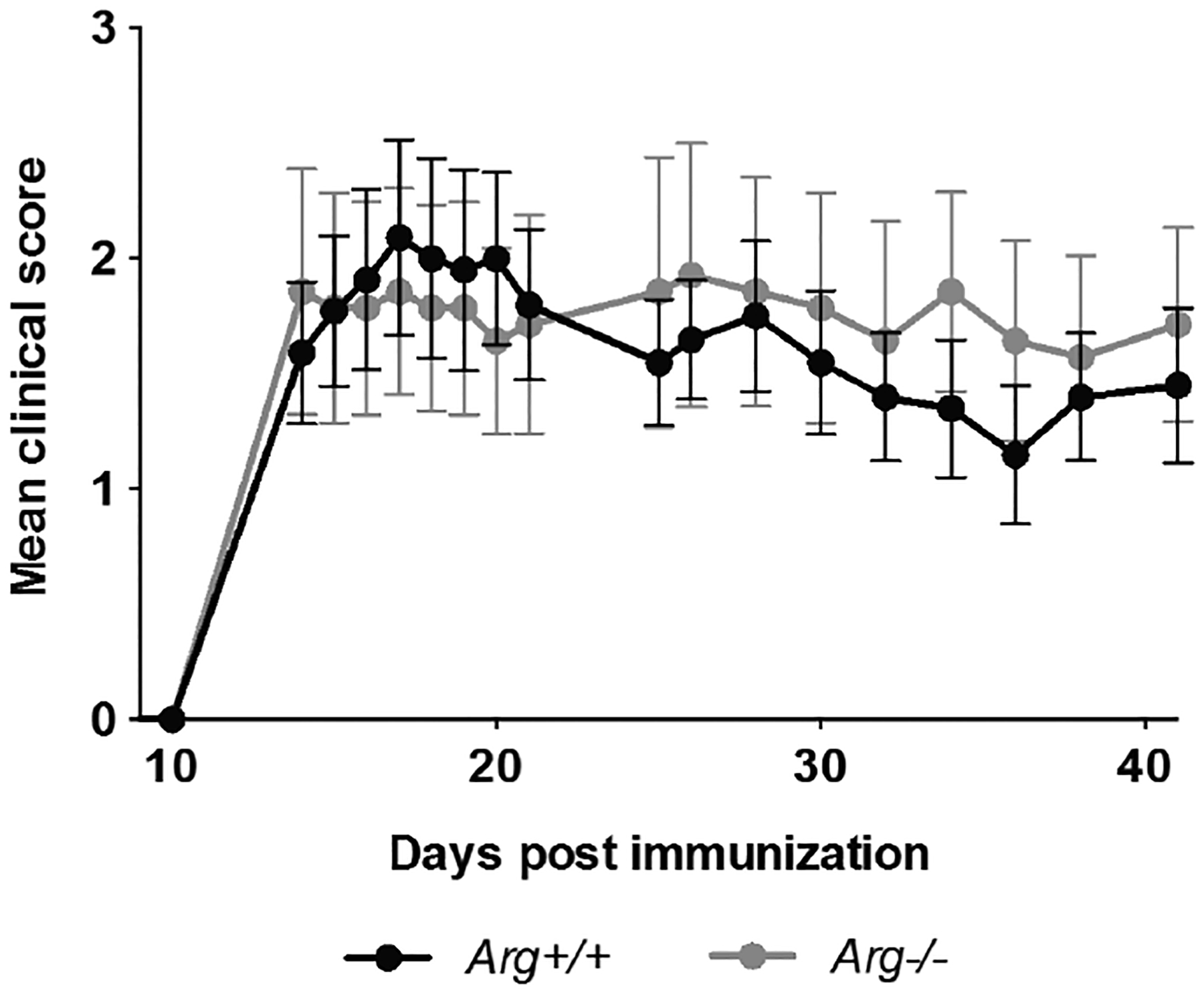
Disease development in Arg^+/+^ (n=11) and Arg^−/−^ (n=7) mice immunized with MOG_35–55_. Data represent mean clinical score ± SEM.

Arg^+/+^ and Arg^−/−^ mice were immunized with the MOG35–55-peptide for EAE induction. No difference in disease onset, incidence, and severity was observed between Arg^+/+^ and Arg^−/−^ mice (Figure 1). Both Arg^+/+^ and Arg^−/−^ mice developed progressive EAE, indicating that absence of Arg does not influence EAE susceptibility in the C57BL/6 background strain upon immunization.

### Lymphocyte activation in Arg^−/−^ mice

**Figure 2:**
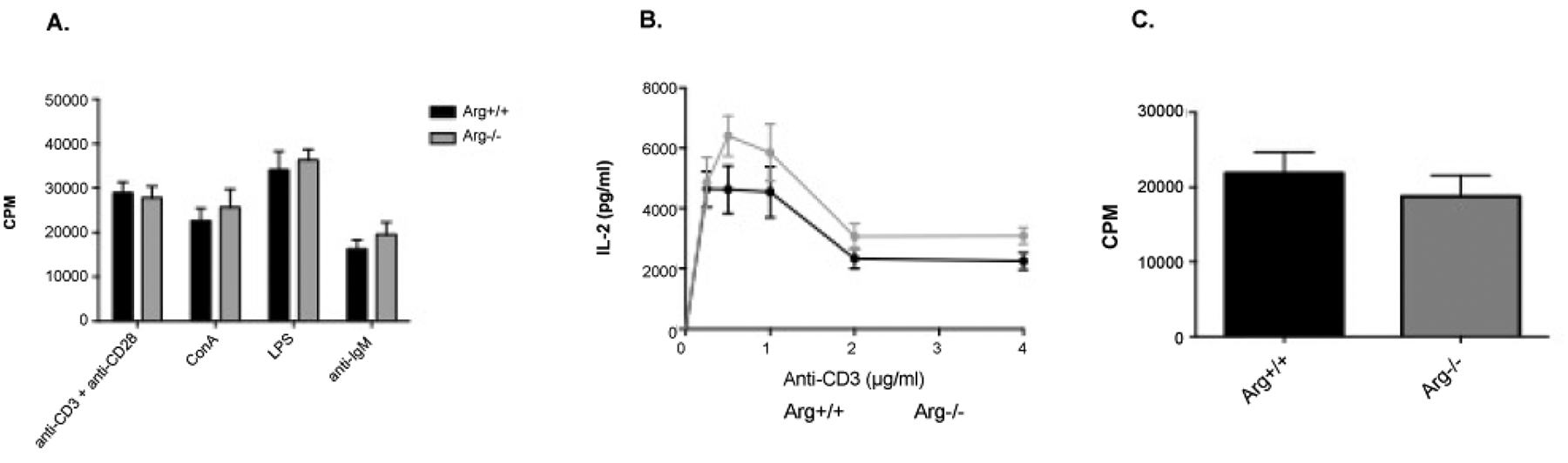
Proliferation and IL-2 production by splenic lymphocytes from naive Arg^+/+^ and Arg^−/−^ mice. (A) Cells were stimulated *in vitro* with anti-CD3 (2 μg/ml)/anti-CD28 (3 μg/ml), ConA (2 μg/ml), LPS (10 μg/ml), and anti-IgM (40 μg/ml), respectively, for 72 hours. Results are based on triplicate measurements from 6 (*Arg*^+/+^) and 7 (*Arg*^−/−^) mice. Data represent mean CPM ± SEM. (B) Cells were stimulated *in vitro* with different concentrations of anti-CD3 and a fixed concentration of anti-CD28 (3 μg/ml) for 48 hours. Results are based on data from 6 (*Arg*^+/+^) and 7 (*Arg*^−/−^) mice. (C) Proliferation of splenic lymphocytes from MOG35–55-immunized Arg^+/+^ and Arg^−/−^ mice. Cells were stimulated *in vitro* with anti-CD3 (2 μg/ml) and anti-CD28 (3 μg/ml) for 72 hours. Results are based on data from 7 (*Arg*^+/+^) and 7 (*Arg*^−/−^) mice and triplicate measurements. Data represent mean CPM ± SEM.

In order to study the role for Arg in lymphocyte activation, the proliferative response of *in vitro* stimulated splenic lymphocytes was measured. No difference in the response to anti-CD3/anti-CD28, ConA, LPS or anti-IgM stimulation was observed between lymphocytes from naive Arg^+/+^ and Arg^−/−^ mice (Figure 2A). In addition, IL-2 was produced to the same levels from *in vitro* stimulated splenic lymphocytes of naïve Arg^+/+^ and Arg^−/−^ mice upon anti-CD3 and anti-CD28 stimulation (Figure 2B). Furthermore, the *in vitro* proliferative response of splenic T-cells from MOG_35–55_-immunized Arg^+/+^ and Arg^−/−^ mice was not influenced by the Arg deficiency (Figure 2C). These results show that loss of Arg has no influence on lymphocyte activation in naive and immunized mice upon *in vitro* stimulation.

### Lymphocyte numbers in Arg^−/−^ mice

**Figure 3:**
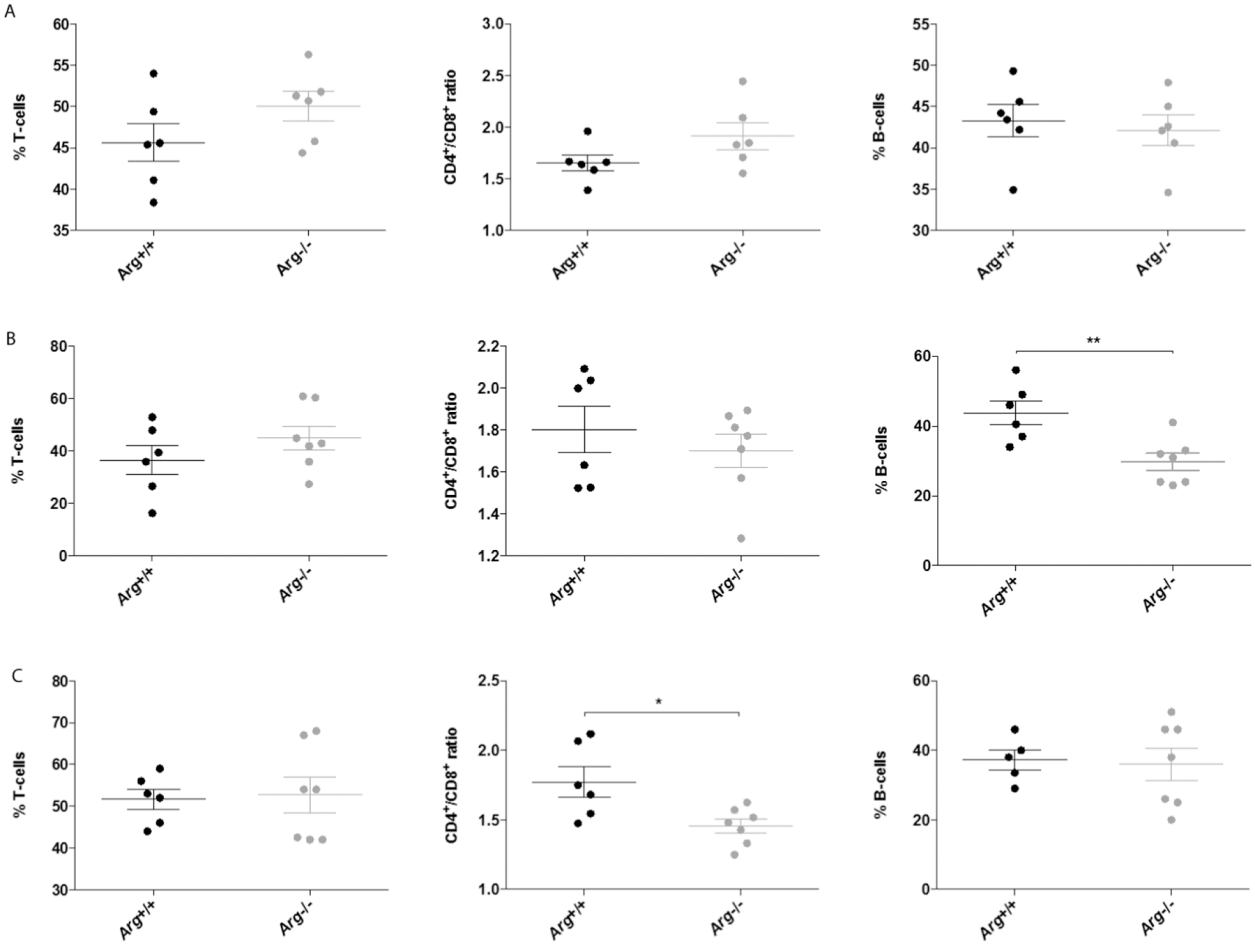
Flow cytometry data showing % T-cells (sum of % CD4^+^ and CD8^+^ lymphocytes), CD4^+^/CD8^+^ ratio, and % B-cells (CD19^+^ lymphocytes) in spleen from (A) naive and (B) MOG_35–55_ immunized Arg^+/+^ and Arg^−/−^ mice. (C) Corresponding flow cytometry data obtained from inguinal lymph nodes from MOG_35–55_ immunized Arg^+/+^ and Arg^−/−^ mice. Data present mean % ± SEM. *p<0.05; **35–55 p<0.01.

The lymphocyte compartment in spleen and lymph nodes from Arg^+/+^ and Arg^−/−^ mice was analyzed by flow cytometry. No differences in the relative numbers of splenic T- and B-cells were observed between naive Arg^+/+^ and Arg^−/−^ mice (Figure 3A). Neither did loss of Arg alter the CD4^+^/CD8^+^ T-cell ratio. In contrast, a significant reduction (p=0.0082) in the percentage of B-cells was observed in the spleen of Arg^−/−^ mice immunized with MOG, when compared to Arg^+/+^ littermates (Figure 3B). No difference in B- and T-cell percentages was found in inguinal lymph node samples from MOG_35–55_ immunized mice, but the CD4^+^/CD8^+^ T cell ratio was significantly lower (p=0.035) in Arg^−/−^ mice compared to Arg^+/+^ mice (Figure 3C). Taken together, these results show that loss of Arg has no influence on the relative T- and B-cell number in naïve mice, but upon immunization with MOG_35–55_, the relative B-cell number is significantly reduced in the absence of Arg.

## Discussion

The genotype ratio of the progeny from the heterozygous Arg^+/−^ breeding revealed that only 10 % of the pups had the Arg^−/−^ genotype (Table 1). This is lower than previously reported for heterozygous breeding [3], and could be a result of differences in genetic background [21]. The low ratio of pups with the Arg^−/−^ genotype suggests that Arg deficiency results in increased embryonic mortality, and demonstrates the importance of the Arg kinase in essential cellular signaling events during development.

Abl kinases are known to play an important role as signal transducers downstream of both T- and B-cell receptors [13,14,22], and could therefore be a possible target in the signaling cascades leading to an immune response against self-proteins. In combination with previous research results, which reveal an ameliorating effect of Abl kinase inhibition for EAE development [18,20], studies of Arg deficient mice in the EAE model, contribute with novel knowledge about the role of the Abl kinases for disease susceptibility and immune cell activation.

We hypothesized that loss of Arg would result in an altered autoimmune response upon EAE-induction. In contrast, our results demonstrate that mice deficient for Arg develop EAE to the same extent as their Arg sufficient littermates upon induction with MOG peptide (Figure 1). Whether this could be a result of overlapping effects of the two Abl kinases, and Abl activity being regulated in response to loss of Arg, is still to be investigated. The N-terminus of the Abl kinases, comprising the SH3 and SH2 domains, which are responsible for establishment of protein-protein interactions [23], and the catalytic kinase activity, are highly similar in sequence and structure [5]. It is therefore reasonable to hypothesize that Abl and Arg take part in the same signaling cascades in immune cell activation, and that Abl activity therefore can make up for the absence of Arg in some cellular signaling events. This is supported by studies in conditional knock-out mice, where loss of both Abl kinases results in pronounced reduction in T-cell number and activation, in contrast to single Abl^−/−^ or Arg^−/−^ knockouts, where only a minor reduction in T-cell activation is observed [13].

Herein presented results show no significant difference in lymphocyte proliferation or IL-2 production between Arg^+/+^ and Arg^−/−^ mice, when stimulated *in vitro* with T- and B-cell stimulating agents (Figure 2). This is in line with other studies showing that presence of both Abl kinases is necessary for full T-cell activation, and that the absence of either Abl or Arg alone only reduces the proliferative response to a minor degree at low concentrations of anti-CD3, when stimulated *in vitro* [13].

Studies of the impact of Arg deficiency for the distribution of lymphocytes in spleen and lymph nodes revealed a significant reduction in the number of splenic B-cells in MOG_35–55_ immunized Arg^−/−^ mice (Figure 3B). No similar difference was found between naive Arg^+/+^ or Arg^−/−^ mice, neither was the relative number of B cells reduced in lymph nodes of immunized mice. The reason for the difference in relative B-cell numbers between spleen and lymph nodes of immunized mice cannot be explained from the present data. It has been shown that the expression of Arg is higher in mature B-cells compared to early stage B-cells [17]. The final maturation of B cells occurs in spleen [24,25] and the lack of Arg could, potentially, lead to a compromised expansion of mature B cells upon immunization. The marginal zone B-cell population is, in the mouse, present only in spleen [26–28]. The level of Arg expression in this cellular compartment is not known, but an effect on marginal zone B-cells due to Arg deficiency, might explain part of the difference between the B cell levels in spleen and lymph nodes of immunized mice.

Furthermore, our results show that the CD4^+^/CD8^+^ ratio in lymph node lymphocytes from MOG-induced Arg^−/−^ mice is significantly reduced compared to WT littermates (Figure 3C). Together with the observation of decreased relative B-cell numbers in the spleen of Arg^−/−^ immunized mice, this suggests that absence of Arg plays a role for the regulation of cell numbers in different lymphocyte populations during activation of the immune system.

In this study we have not directly addressed the role for Arg as a key regulator of actin organization. Loss of Arg might result in altered regulation of cellular events involving spatiotemporal actin cytoskeletal dynamics, influencing lymphocyte migration and adhesion. The importance of these activities for the normal immune response remains to be investigated. From the present study, we conclude that the absence of Arg does not play a role for the induction of T-cell mediated autoimmunity in the CNS.

## Conclusion

Here we have shown that Arg-deficient mice develop EAE with similar incidence and progression as observed for WT littermates, but that the relative number of splenic B-cells is significantly reduced in Arg^−/−^ mice after immunization with MOG peptide. Furthermore, we observe a skewed frequency of born Arg^−/−^ pups, and suggest that this demonstrates an essential role for Arg during embryonic development.

## Figures and Tables

**Table 1:** Genotype frequency of born pups from breeding of Arg^+/−^mice.

Genotype	Born pups	% of total^a^	% expected frequency
**Arg** ^+/+^	70	34	25
**Arg** ^+/−^	117	56	50
**Arg** ^−/−^	21	10	25

a % of total number of born pups

## Abbreviations

EAE: Experimental Autoimmune Encephalomyelitis
Arg: Abelson Related Gene
MOG: Myelin Oligodendrocyte Glycoprotein
F-actin: Filamentous actin
ConA: Concanavalin A
LPS: Lipopolysaccharide
